# Selective Arcing Electrostatically Eradicates Rice Weevils in Rice Grains

**DOI:** 10.3390/insects12060522

**Published:** 2021-06-04

**Authors:** Koji Kakutani, Yoshihiro Takikawa, Yoshinori Matsuda

**Affiliations:** 1 Pharmaceutical Research and Technology Institute and Anti-Aging Centers, Kindai University, Osaka 577-8502, Japan; kakutani@kindai.ac.jp; 2 Plant Center, Institute of Advanced Technology, Kindai University, Wakayama 642-0017, Japan; 3 Laboratory of Phytoprotection Science and Technology, Faculty of Agriculture, Kindai University, Nara 631-8505, Japan; ymatsuda@nara.kindai.ac.jp

**Keywords:** electric field, transient electric current, sound intensity, stored product, pest, habitual behavior, behavioral analysis

## Abstract

**Simple Summary:**

Rice stored in warehouses is frequently attacked by rice weevils, which must therefore be controlled. We developed an electrostatic method as an alternative to pesticides. An arc discharge exposer (ADE) kills insects entering an electric field. The apparatus has pairs of identical metal plates, one of which is linked to a voltage generator for negative charging, the other is grounded. A −7 kV electric field forms between the two plates. When an insect enters the electric field it is arced by the charged plate and killed. We combined this method with a technique that lures insects hiding in rice grains. When a vessel containing rice and insects was rotated and then returned to the initial position, the weevils climbed up the vessel walls and entered the ADE. Dead insects were collected to minimize rice contamination. This method efficiently killed pests in stored rice.

**Abstract:**

We developed an arc discharge exposer (ADE) that kills rice weevils nesting in dried rice. The ADE features multiple identical metal plates, half of these are linked to a voltage generator and the others are grounded. The plates were arrayed in parallel and an electric field formed between them. Any insect entering the field was arced from the negatively charged plate and killed. The ADE was placed on a vessel containing pest-infested rice grains; the insects were lured out of the grains by mechanically vibrating the vessel. When rice grains move, insects tend to climb upward, thus, the weevils were effectively removed. Our electrostatic apparatus is easy to construct and could be used to control pests in stored rice.

## 1. Introduction

Rice has been organically cultivated without pesticides (herbicides, fungicides, and insecticides). However, harvested rice is commonly infested with cereal insect pests, particularly the rice weevil (*Sitophilus oryzae* Linnaeus) (Coleoptera: Curculionidae) which destroys warehoused grains, seeds and grain products [1]. Adult weevils live for 4–5 months and each female lays 300–400 eggs during this period. The female uses her strong mandibles to chew holes in grain kernels, in which she deposits single eggs and seals the holes with a gelatinous fluid. Larvae develop within the grain kernels, eat the grain, and frequently cause near-complete grain destruction. Adults disperse by crawling, allowing infestations to spread to neighboring areas [2]. In Japanese warehouses, rice polishing, packing into paper bags, and repacking for storage are conducted in the same space. Frequently, powdered grains lie scattered on the floor and attract weevils. Pest entry to the warehouse should, ideally, be prevented. Also, sanitation (especially before the delivery of new grain) is essential. However, infestations remain frequent and fumigation is often inevitable [3,4,5,6]. This is both difficult and costly; fumigants are very toxic to humans and must be applied skillfully. Thus, we developed an electrostatic device to selectively kill rice weevils. Electric fields have been used to manage insects, pathogens, and weeds as alternatives to pesticides, because of pesticide resistance and the public demand that pesticide use is minimized [7]. An electrostatic field is a space surrounding an electric charge within which a perceptible force can be imparted to another electric charge [8]. Typically, a negatively or positively charged conductor is placed some distance from a grounded conductor [9]. The larger the voltage, the more powerful the electric field because of the greater potential difference. When an insulated conductor is used, a non-discharging electric field (i.e., no electric current flows between the two conductors) is produced [7]. Conversely, a non-insulated charged conductor produces a discharge-generating electric field in which coronal and arc discharges occur depending on the distance and potential difference between the two conductors [7].

Pest control methods using static electric fields exploit the force generated to attract insects entering the fields [10,11,12,13]. Methods using dynamic electric fields pass electricity through insects causing electrocution/severe injury [14,15]. Here, we used a dynamic electric field to kill rice weevils in rice grains from storage bags. We modified a previous method [15] to construct a novel arcing device.

This electrostatic method was combined with a simple technique that lures adult weevils hiding among rice grains. We found that adult rice weevils released onto dried rice in a vessel immediately dug into the spaces among the grains and did not emerge if the vessel was stationary. However, when the vessel was tapped, vibrated, shaken, or rolled (thus moving the grains), insects near the vessel quickly climbed up the wall to the top, walked around, and hid again in the absence of an additional stimulus. Insects that were not close to the wall tried to move horizontally toward the wall and then climb up. However, such horizontal movement through the spaces among grains was difficult, because of the weight of the grains. It was easier to move vertically along the wall than within the rice. The behavior described above was highly reproducible.

Here, we studied weevil behavior in small cells with various dimensions; a box size allowing all insects to reach the top surface after an external stimulus was sought. We used an arc discharge exposer (ADE) to then kill the insects and used the system as a preceding step for pest-free packing.

## 2. Materials and Methods

### 2.1. Insect Species

Adult rice weevils (*S. oryzae*) were purchased from Sumika Technoservice (Hyogo, Japan) November 2020 and reared on rice grains in plastic containers (diameter: 20 cm; height: 10 cm) in a growth chamber at 25 ± 2 °C and 60 ± 5% relative humidity (RH) for two months. Newly emerging adults were collected using an insect aspirator (Wildco, Yulee, FL, USA). The average body size (mean length from head to wingtip of 20 adults) was 2.51 ± 0.24 mm.

### 2.2. Habitual Behavior

We prepared polypropylene cells with one open side (1 × 1 × 1, 1 × 1 × 2, 1 × 1 × 3, 1 × 1 × 5, 2 × 2 × 2, 2 × 2 × 3, 2 × 3 × 5, 3 × 3 × 3, 3 × 3 × 5 and 5 × 5 × 5 cm^3^; 1^2^S-1H-, 1^2^S-2H-, 1^2^S-3H-, 1^2^S-5H-, 2^2^S-2H-, 2^2^S-3H-, 2^2^S-5H-, 3^2^S-3H-, 3^2^S-5H-, 5^2^S-5H- cells, respectively). For example, the 1^2^S-1H cell had a base area of 1 cm^2^ and a height of 1 cm. The details are listed in Table S1. Cells of the same dimensions were pasted together on a polypropylene board (30 × 30 cm^2^). Rice grains (100 g) containing 10 adult weevils were used to fill the cells on the board. The plate with the cells was placed on a table vibrator (AS ONE, Osaka, Japan) and shaken for 10 s at 60 vibrations/s. After 5 min, weevils that emerged from the grain were counted to identify cells in which all insects were lured out.

### 2.3. Construction of the ADE

**Figure 1:**
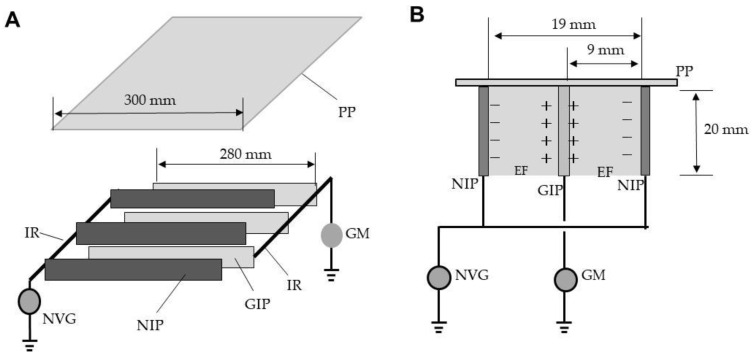
The arc discharge exposer (ADE) (**A**), in cross-sectional view (**B**). Abbreviations: PP, polypropylene plate (insulator); IR, iron rod; NVG, negative voltage generator; NIP, negatively charged iron plate (conductor); GIP, grounded iron plate (conductor); GM, galvanometer; EF, electric field.

Figure 1A shows the ADE. Several iron plates (20 × 280 mm^2^; 1-mm-thick) were arrayed in parallel at intervals of 19 mm and welded to an iron rod. For each pair of plates, one was linked to a direct-current voltage generator (maximum current, 10 mA) (Max-Electronics, Tokyo, Japan) and negatively charged at different voltages (negatively charged iron plate; NIP), while the other was grounded (grounded iron plate; GIP). The plates were alternately arranged in parallel at intervals of 9 mm along a comb-shaped polypropylene spacer attached to a polypropylene board (30 × 30 cm^2^), and fixed with a polypropylene frame. Each NIP created a positive change on the facing surface of the GIP [16], and an electric field thus formed (Figure 1B). Free electron transfer from the NIP to the GIP via arcing was detected using a galvanometer (PC7000; Sanwa Electric Instruments, Tokyo, Japan) placed on the ground line.

### 2.4. Insect-Mediated Arcing and Voltage-Dependent Eradication

**Figure 2:**
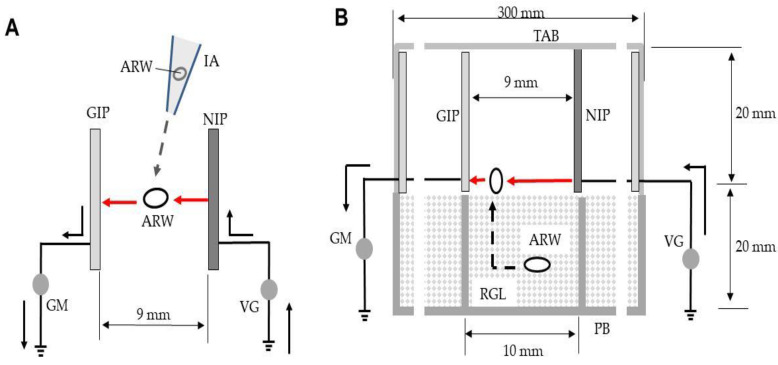
(**A**) Arcing of rice weevils blown into the space between the opposite poles (NIP and GIP) of the ADE. (**B**) Arcing of weevils emerging from rice into the electric field. The solid arrow represents electron movement in the absence of arcing and the red arrow denotes movement during arcing. The dotted arrow is the path taken by the insects. Abbreviations: IA, insect aspirator; ARW, adult rice weevil; GIP, grounded iron plate (conductor); NIP, negatively charged iron plate (conductor); GM, galvanometer; VG, voltage generator; TAB, transparent acrylic box; RGL, rice grain layer; PB, polypropylene box.

In preliminary experiments, the ADE was negatively charged from −1 to −12 kV to determine the lowest voltage that produced arcing in the absence of weevils. Mechanical discharge was absent from −1 to −10 kV. Next, adult rice weevils were blown into the spaces between the plates, using an insect aspirator, to determine the voltage that triggered arcing from the NIP to the insect (Figure 2A). Twenty insects were tested at each voltage. The loudness of each discharge was measured in decibels (Sound-level meter C-323; Sato Tech, Kanagawa, Japan) as described previously [17]. The sound profile was recorded by a spectrum analyzer integrated into the sound-level meter. Simultaneously, the current profile was recorded using a current detector integrated into the galvanometer. Insects exposed to arcing were flung out of the ADE, and then collected and examined. Survival was assessed 6 h later. Insects walking at this time were considered to be alive. All experiments were video-recorded.

Next, the ADE was placed inside a transparent acrylic box (bottom area, 30 × 30 cm^2^; wall height, 2 cm) with a lid that prevented insect expulsion (Figure 2B). This box was placed upside down onto 1^2^S-2H cells on the plate mentioned above; the latter cells were filled with rice containing weevils. The entire apparatus was vibrated as described earlier; the insects moved to the top of the rice and were arced from the nearest NIP on emergence (Figure 2B). The ADE voltage was −7 to −10 kV. All insects were then collected. Twenty insects were exposed to each voltage. Sounds and electric currents were measured.

### 2.5. Construction of a Practical ADE-Integrated Insect Eradicator (A-IE)

**Figure 3:**
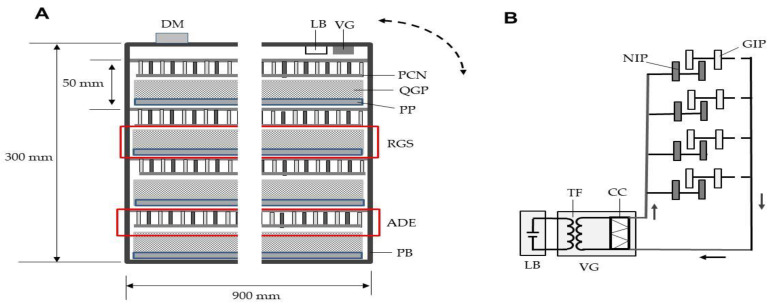
(**A**) The ADE-integrated insect eradicator (A-IE). Four ADEs were fixed in a polypropylene box. Four rice grain servers (a polypropylene board onto which a quadrate-grid plate was pasted and filled with insect-containing rice grains) were slid into the box beneath the ADEs. The ADEs were charged to −7 kV using a voltage generator powered by a rechargeable lithium battery. The A-IE was rotated through 90° and then returned to the original position, causing the insects to climb to the top of the rice. The dotted arrow represents A-IE rotation. (**B**) Schematic of the non-grounded A-IE circuit. The arrow indicates the direction of electron movement. Abbreviations: DM, directional microphone; LB, lithium battery; VG, voltage generator; RGS rice grain server; ADE, arc discharge exposer; PCN, polyvinyl chloride net; QGP, quadrate-grid plate; PP, polypropylene plate; PB, polypropylene box; NIP, negatively charged iron plate; GIP, grounded iron plate; TF, transformer; CC, Cockcroft circuit.

Figure 3A shows the A-IE box containing four ADEs and four rice grain servers (RGSs). The NIPs and GIPs of the ADEs were installed vertically on a polypropylene board (90 × 90 cm^2^), and the ends were covered with soft polyvinyl chloride nets (90 × 90 cm^2^; mesh size 1.5 mm; net string diameter 0.1 mm). The ADEs were arrayed in parallel at 5-cm intervals. Each RGS featured a quadrate-grid plate (90 × 90 cm^2^; one grid volume, 1 × 1 × 2 cm^3^; 6400 grids per plate) attached to a polypropylene board of the same size. Rice grains containing 20 weevils were evenly distributed among all grid holes on the RGSs. The RGSs were slid into the box below the ADEs.

The NIPs and GIPs of all ADEs were linked together, and to a single voltage generator, forming a non-grounded circuit (Figure 3B). The NIPs were charged to −7 kV. In a non-grounded circuit, free GIP electrons are supplied directly to NIPs. Therefore, the ADEs did not require a ground line. The voltage generator had a rechargeable lithium battery (12 V).

The A-IE was rotated through 90°; the insects moved to the top of the grain (Figure 3A). Transient electric currents and sounds associated with insect arcing were recorded. The A-IE featured a directional microphone that transmitted sounds via wireless to the sound-level meter.

### 2.6. Statistical Analysis

All experiments were repeated five times; all data are presented as means with standard deviations. Tukey’s test, using EZR software (ver. 1.54; Jichi Medical University, Saitama, Japan), was used to detect differences among the various conditions.

## 3. Results and Discussion

### 3.1. Habitual Behavior of Adult Rice Weevils

Habitual pest insect behavior must be understood when establishing control methods. Vinegar flies and cigarette beetles [18], as well as whiteflies [19] and rice weevils [20], avoid entering static electric fields; such fields repel insects [21]. Obviously, the insect must perceive the field. Newland et al. [22] found that cockroaches detected electric fields with their antennae, deflecting them from the attractive force toward the negative electrode. Nonomura et al. [19] and Matsuda et al. [20] found that insects inserted their antennae into electric fields formed between opposite poles. This constituted “searching” behavior; the insects did not enter the fields. Antennae appear capable of detecting repulsive electric force; antennal electrons are disrupted by electrostatic induction [22].

We studied the habitual behavior of weevils nesting in dried rice. We consistently found that weevils near a vessel wall quickly climbed the wall (up to 6 cm in height) to emerge on top of the rice if the vessel was tapped, vibrated, shaken, or rotated (Video S1A). Here, we used a controllable table vibrator. We consistently found that adult rice weevils near the vessel wall quickly climbed up the wall to the top, when the vessel was tapped, vibrated, shaken or rolled. On the other hand, insects that were not close to the wall tried to move horizontally toward the wall and then climbed up after the external stimulus was added to the vessel.

However, some insects were immobilized by larger amounts of packed rice and did not reach the surface (Video S1B). Upper grains compacted lower grains so the space between grains required for insect movement was not available. Such insects survived for at least 1 month.

**Figure 4:**
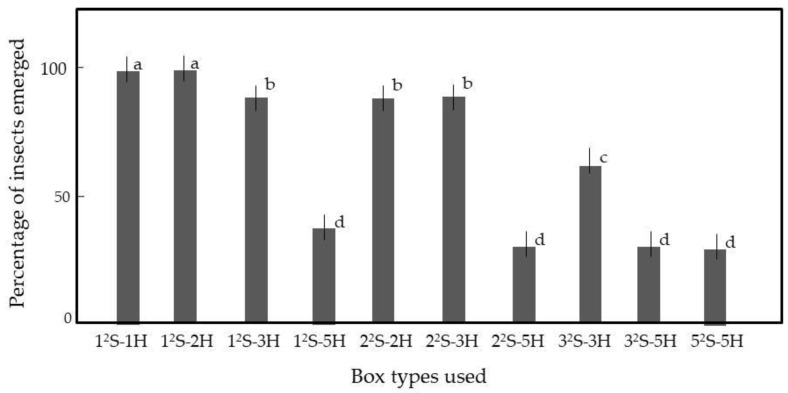
Emergence of adult rice weevils from rice grains in cells of various sizes. Rice grains were mixed with 50 weevils. Table S1 provides the box details. Means and standard deviations of five replicates are shown. Letters (a–d) on a column indicate significant differences (*p* < 0.05), as revealed by the Tukey method.

We required that all insects in rice should move to the top when the rice was vibrated. We added insect-containing rice to a variety of cells to examine the effects of the spaces between the grains on insect movement. Most importantly, it was essential that an external stimulus (in this case, vibration) should be spread equally to the rice grains in all cells. For this purpose, all cells were pasted on the board. Figure 4 shows the 1^2^S-1H and 1^2^S-2H cells from which all insects emerged on top of the rice within 5 min of vibration, indicating that the insects could move to the walls and then climb up. We used the 1^2^S-2H-cell for the following experiment, as it contained more rice than the 1^2^S-1H-cell (Table S1).

### 3.2. Insect-Mediated Arcing

**Figure 5:**
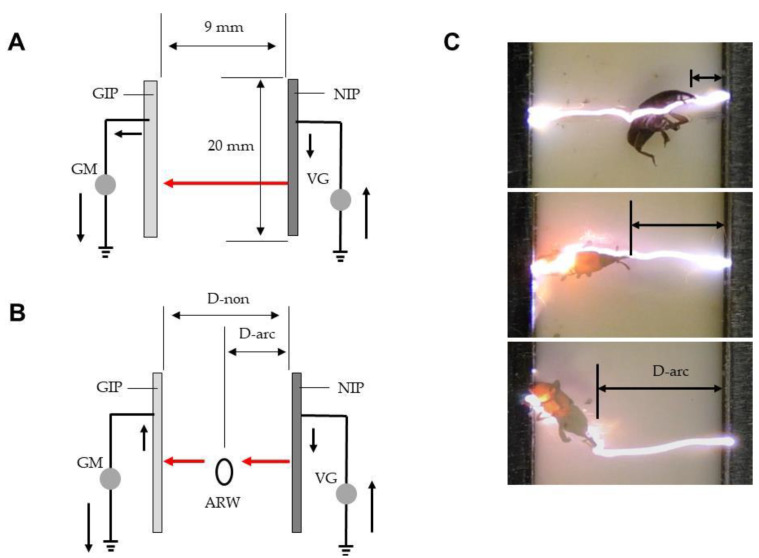
(**A**) Schematic representation of mechanical (arc) discharge between opposite poles (NIP and GIP). (**B**) Schematic representation of insect-mediated arcing between the poles of the ADE. (**C**) Rice weevils exposed to arcing at various distances from the NIP. The solid arrow represents the movement of electricity and the red arrow denotes the aerial movement of electricity via arcing. Abbreviations: GIP, grounded iron plate (conductor); NIP, negatively charged iron plate; GM, galvanometer; VG, voltage generator; ARW, adult rice weevil; D-non, the distance associated with no arcing; D-arc, the distance from the NIP at the time of arcing.

A discharge is an electric current generated between opposite poles by the dielectric breakdown of gases in the electric field, according to the potential difference between the poles [16]. We created a circuit in which free electrons moved from ground to ground via discharge (Figure 5A). High voltages produced by a Cockcroft circuit [18] in the voltage generator were used to electrify both electrodes; electrons were added to the NIP from the ground and pushed out of the GIP to the ground. The flow of electrons on a NIP is impeded by the electric resistance of air between the poles; pole separation was fixed in our ADE. NIP discharge depends on the voltage applied. As the voltage applied to the NIP increased, mechanical discharge was evident at > 10 kV (Figure 5A). Thus, at ≤ 10 kV, mechanical discharge was absent.

Figure 5B shows that when an insect enters the electric field between the NIP and GIP at any point, it becomes an intermediate pole and receives an arc from the NIP because it is conductive. The electrons are then transferred to the GIP; this is termed insect-mediated arc discharge [19]. The discharge is transient but can kill the insect; the higher the voltage, the higher the mortality rate. We identified the highest voltage associated with no mechanical arcing from the NIP in the absence of an insect, the voltage range triggering insect-mediated arcing, and the voltage with the highest mortality rate. We also explored the potential of the A-IE for practical application.

A conductor entering the space between opposite poles can receive electrons from a NIP and donate them to a GIP. Insects are bio-conductors (Figure 5B). Several studies [20,21,22,23,24] found that the outer protective cuticle of many invertebrates is efficiently electrified. Also, insect body-water conducts electricity [25]; we exploited insect conductivity.

**Figure 6:**
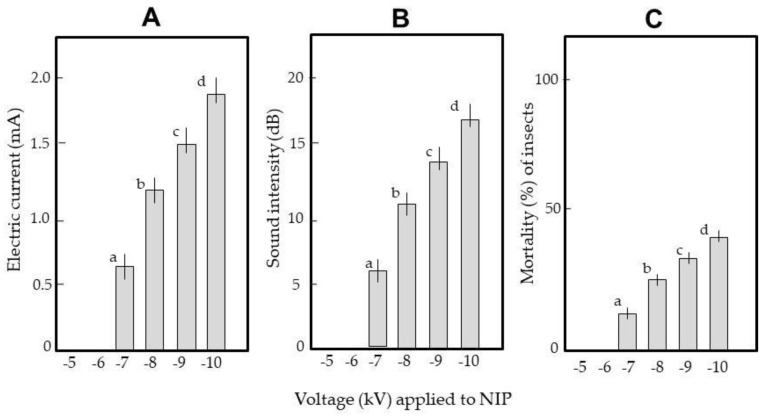
Generation of transient electric currents (**A**) and arcing sounds (**B**), and the mortality (**C**) of weevils arced in the electric field of the ADE. Insects were blown into the space between the NIP and GIP of an ADE negatively charged at different voltages. Twenty insects were tested at each voltage; the means and standard deviations of five replicates are shown. Letters (a–d) on a column indicate significant differences (*p* < 0.05), as revealed by the Tukey method.

First, weevils were blown into the space between the NIP and GIP of ADEs charged with different voltages. Figure 6 shows the transient electric current (A), sound intensity (B), and mortality rate (C) at each voltage. Figure 6A shows that the weevils were conductive; current flowed through the insect body during arcing. The current developed at −7 kV and became higher as the voltage increased. The arc discharge sound exhibited similar behavior (Figure 6B). The sound is a sonic boom caused by the shock wave of high-speed electrons; the intensity reflects the wave strength. The insects were blown out of the apparatus (Video S2A). However, mortality was < 50% even at −10 kV (Figure 6C). Sound was taken to indicate arcing.

Next, the ADE was installed in a box; arced insects were not forced out of the electric field. We also examined whether we could exploit habitual insect behavior to kill them. The ADE was charged to −7 kV. At this voltage, no electrons were transferred from the NIP to rice, which is an insulator [15]. In all of 50 replicates, insects emerged from the rice after vibration and were arced, i.e., flung toward the GIP or ceiling and wall, and bounced back for additional arcing (Video S2B); eventually arcing stopped. All insects were killed.

**Figure 7:**
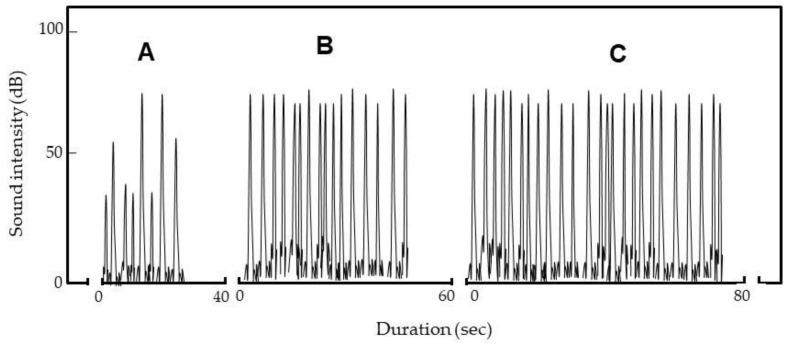
Spectrograms of sounds generated on arcing of one (**A**), two (**B**) and three insects (**C**) by the ADE (−7 kV). Weevils emerged from rice after vibration and were repeatedly arced.

Figure 7 shows the discharge sounds when one, two, and three insects entered the electric field. For single insects (Figure 7A), the sound intensity increased as the insects were repeatedly arced. The video shows that arcing occurred at various distances from the NIP (Figure 5C). Arcing nearer the NIP was louder than arcing remote from the NIP. As stated above, the insect is an intermediate pole (Figure 5B); the shorter the distance between the insect and NIP, the greater the potential difference [16] and the stronger the arcing.

When several insects entered the electric field, sound intensity did not vary much (Figure 7B,C) because the insect nearest the NIP at any time was selectively arced (Video S2C). The number of sounds (i.e., arcs) increased as the number of insects increased. Eventually, arcing stopped, and all insects died.

Why did arcing stop? Arcing exploits the conductive nature of the insect. Takikawa et al. [25] found that body water explained insect conductivity. Matsuda et al. [17,26] showed that arcing vaporized plant body water by heating, based on the Joule effect [27] and a reduction of conductivity. Artificially dehydrated insects were not arced in an electric field [25]. We suggest that weevils subjected to multiple arcings lost their body water via vaporization, died, and became non-conductive; arcing thus ceased. In fact, in a preliminary experiment, we found that insecticide-killed weevils were arced in an electric field; we plan to explore this further.

### 3.3. Modification of the ADE for Practical Use

In a previous work [15], we first developed an electrostatic apparatus to eradicate rice weevils in rice grains by means of an insect-mediated arc discharge exposure. This apparatus consisted of a pair of oppositely charged metal nets that were vertically arrayed in parallel at a definite interval, and rice grains containing the weevils were dropped down through the space between the nets for selectively arcing to the weevils. Although the weevils were effectively subjected to the arcing, some insects occasionally escaped the arcing and, more seriously, the apparatus was clogged with rice grains that were continuously introduced into the net space. The present apparatus was newly developed to solve this problem.

**Figure 8:**
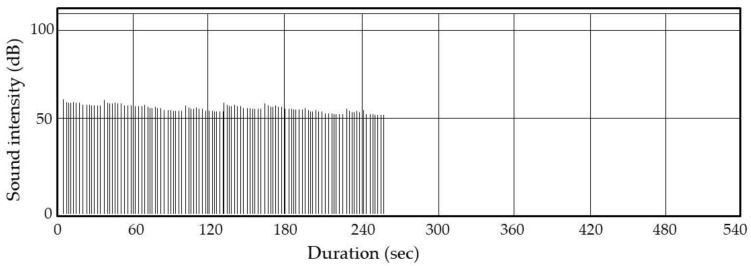
The sound intensities from the A-IE. The signals were transmitted via wireless from a highly directional microphone to the sound analyzer.

We exploited the habitual behavior of weevils (i.e., climbing upward in response to motion) to kill them. For scale-up, we rotated a box through 90°; all insects emerged within 2–3 min. We covered the rice with a net through which insects could pass. Video S3 shows that all insects walked around on the net and were thus readily exposed to arcing. Our A-IE prototype can treat 10 kg of dried rice. Figure 8 shows the result of one of five identical assays using 10 kg of rice grains containing 20 weevils stimulated by reciprocal 90° rotations. All assays yielded the same results. Figure 8 shows that all arcings occurred within the first 4 min. All insects were killed and their body fragments typically were collected between the ADEs and the net. This minimized rice contamination by the fragments. However, we observed that 60% of dead rice weevils lost their legs and/or wings and these detached fragments occasionally fell into the rice grains. Further research will focus on the optimization of the electrostatic conditions that kill the rice weevils without causing dismemberment.

In our laboratory case, rice was grown in our experimental paddy field (approximately 0.3 ha) every year and dried rice grains (1000–1200 kg) were packed for storage after normal procedures of threshing, dehydrating, hulling and polishing. This amount of rice grains could be arcing-treated within 10 h using the present A-IE.

Our method could be used after rice polishing and before packaging. The method may also kill other major stored-product pests, such as the cigarette beetle (*Lasioderma serricorne*), red flour beetle (*Tribolium castaneum*), and Azuki bean weevil (*Callosobruchus chinensis*). In a preliminary study, our method controlled maize weevils (*Sitophilus zeamais*) nesting in stored rice grains. Our preliminary study revealed that the arc-discharge exposure was effective to kill other major stored-product pests, such as the cigarette beetle (*Lasioderma serricorne*), red flour beetle (*Tribolium castaneum*) and Azuki bean weevil (*Callosobruchus chinensis*), and that the present vibration-luring/arcing method was applicable to control maize weevils (*Sitophilus zeamais*) nesting in stored rice grains.

In the present apparatus, two identical iron plates paralleled at a definite interval—one was linked to a negative voltage generator and another to a ground line—which were the core of the structure. Most importantly, the entire surface of the charged iron plate could be a site for arcing. Due to this electrostatic property, any size of plate can be used according to the desired scale of the apparatus. This is advantageous for scaling up the apparatus for practical use.

Habitual pest insect behavior must be understood when establishing control methods. Vinegar flies and cigarette beetles [28], as well as whiteflies [29] and rice weevils [30], avoid entering static electric fields; such fields repel insects [31]. Obviously, the insect must perceive the field. Newland et al. [32] found that cockroaches detected electric fields with their antennae, deflecting them from the attractive force toward the negative electrode. Nonomura et al. [29] and Matsuda et al. [30] found that insects inserted their antennae into electric fields formed between opposite poles. This constituted “searching” behavior; the insects did not enter the fields. Antennae appear capable of detecting repulsive electric force; antennal electrons are disrupted by electrostatic induction [32]. These works were associated with the development of an electric field screen that repels the insects that reached the screen [7]. Additionally, the present work specified the habitual behavior of adult rice weevils in response to rice grain movement, by which the new electrostatic apparatus was developed to kill them in rice grains through the insect-mediated arcing discharge exposure.

## 4. Conclusions

We aimed to protect post-harvest crops from insect pests; the rice weevil is a major problem in rice warehouses. Our electrostatic approach is an alternative to pesticides. Insects were lured out of rice into ADEs, which killed them quickly. The method has practical applications.

## Supplementary Materials

The followings are available online at https://www.mdpi.com/article/10.3390/insects12060522/s1, Table S1: The different boxes used to assess the behavior of adult rice weevils in rice grains, Video S1: (A) Insects climbing up the wall of a vessel that holds rice after vessel vibration. (B) Insects immobilized by larger amounts of rice, Video S2: (A) An adult rice weevil knocked out of the ADE by arcing. (B) An adult rice weevil repeatedly bounced off the GIP of the ADE by multiple arcings. (C) Selective arcing of one of two adult rice weevils simultaneously in the electric field, Video S3: Adult rice weevils emerging from non-covered (A) and net-covered rice grains (B) onto the grid plate of the rice grain server after vibration thereof.
