# Supplementary material for: Selective Arcing Electrostatically Eradicates Rice Weevils in Rice Grains
Source: Insects. 2021 Jun 4;12(6):522. doi: 10.3390/insects12060522 (PMC8228487; doi:10.3390/insects12060522)
Supplement: Supplementary file 1 [file insects-12-00522-s001.zip › Table S1.pdf]

**Table S1**. The different cells used to assess the behavior of adult rice weevils in rice grains

| Cells used | Size of cell | | Volume ($cm^3$) of each cell | Number of cells used [a] | Amounts (g) of rice grains [b] | Number of insects [b] |
|---|---|---|---|---|---|---|
| | base area ($cm^2$) | Height (cm) | | | | |
| I$^2$S-1H | 1 × 1 | 1 | 1 | 900 | 738 | 70 |
| I$^2$S-2H | 1 × 1 | 2 | 2 | 900 | 1476 | 140 |
| I$^2$S-3H | 1 × 1 | 3 | 3 | 900 | 2214 | 220 |
| I$^2$S-5H | 1 × 1 | 5 | 5 | 900 | 3690 | 360 |
| 2$^2$S-2H | 2 × 2 | 2 | 8 | 225 | 1640 | 160 |
| 2$^2$S-3H | 2 × 2 | 3 | 12 | 225 | 2460 | 240 |
| 2$^2$S-5H | 2 × 2 | 5 | 20 | 225 | 3690 | 360 |
| 3$^2$S-3H | 3 × 3 | 3 | 27 | 100 | 2214 | 220 |
| 3$^2$S-5H | 3 × 3 | 5 | 45 | 100 | 3690 | 360 |
| 5$^2$S-5H | 5 × 5 | 5 | 125 | 36 | 3690 | 360 |

[a] Cells of the same size were pasted together onto a polypropylene board (30 × 30 $cm^2$).

[b] Designated amounts of rice grains (*Oryza sativa* L., Yumepirica; short-grain type) containing the specified number of weevils were distributed evenly among all cells on a plate.
